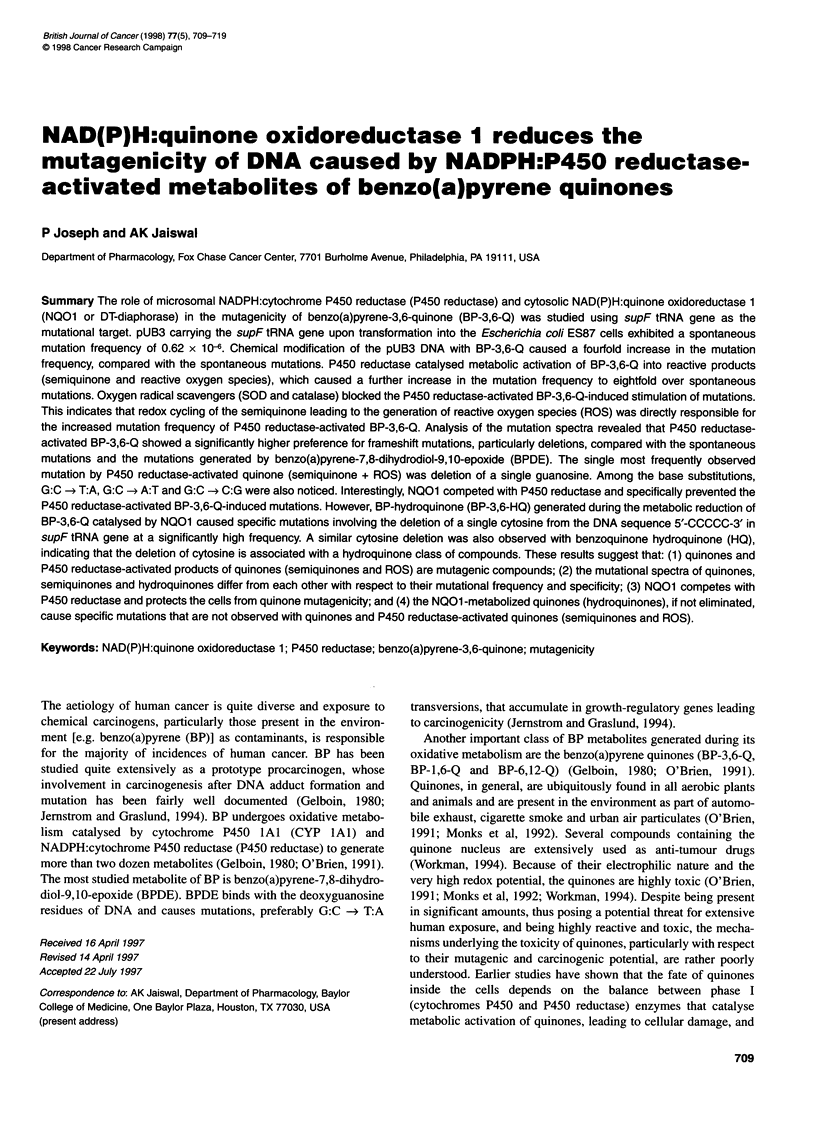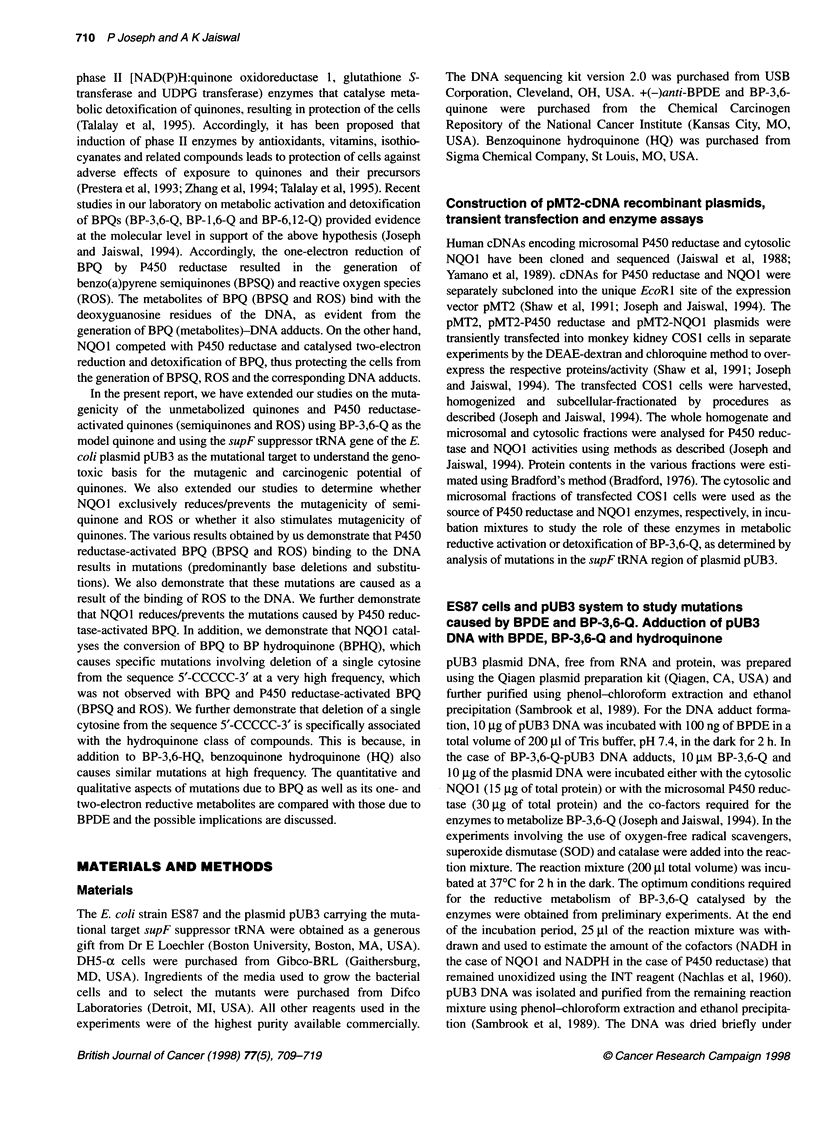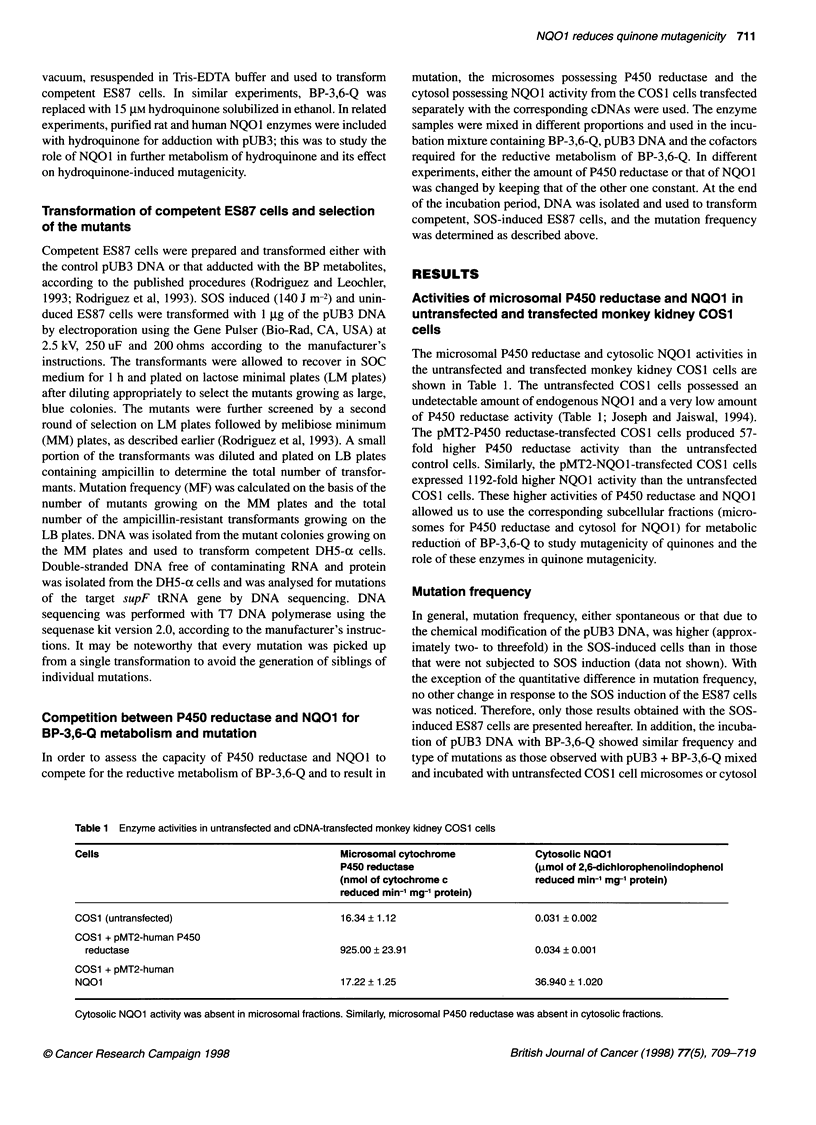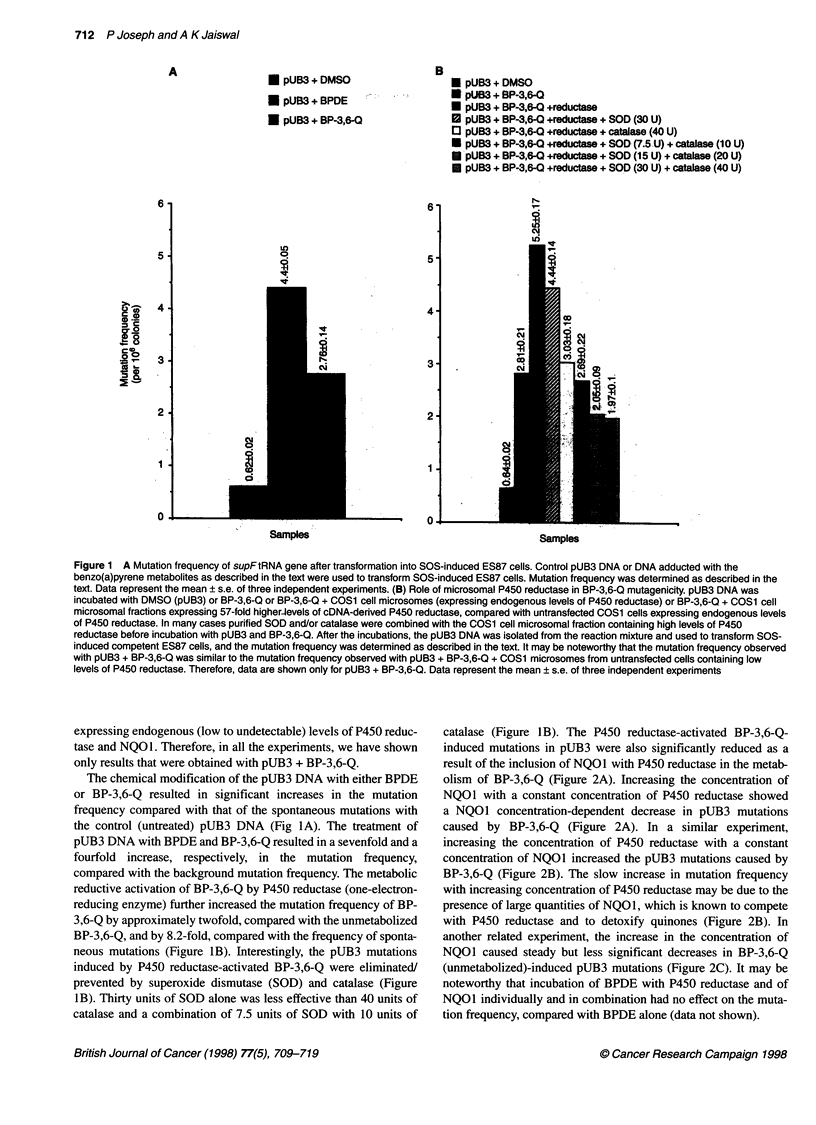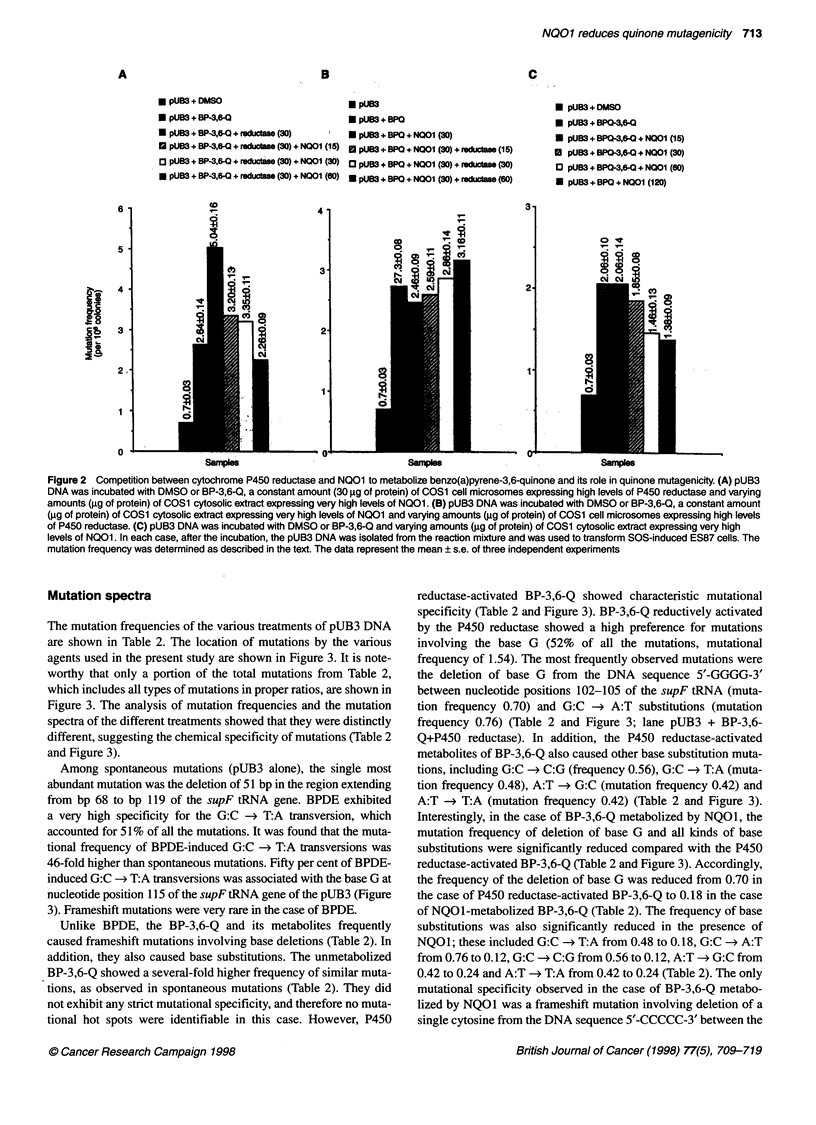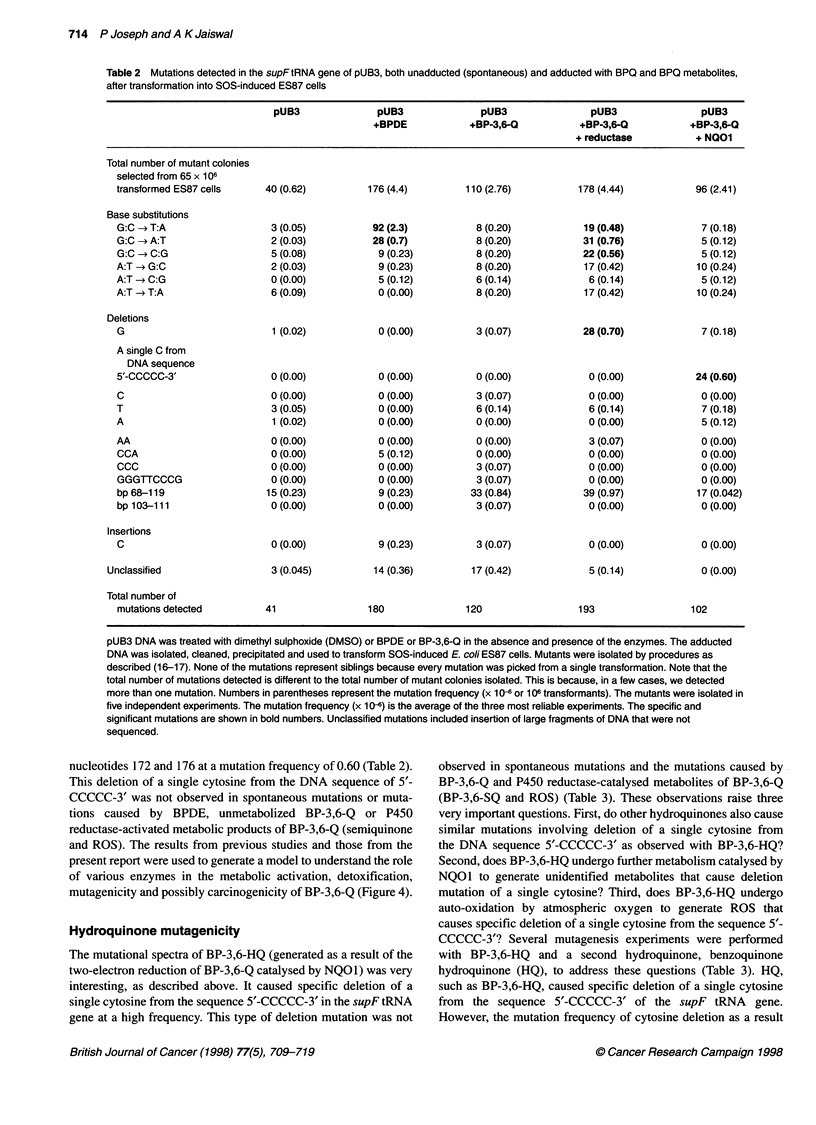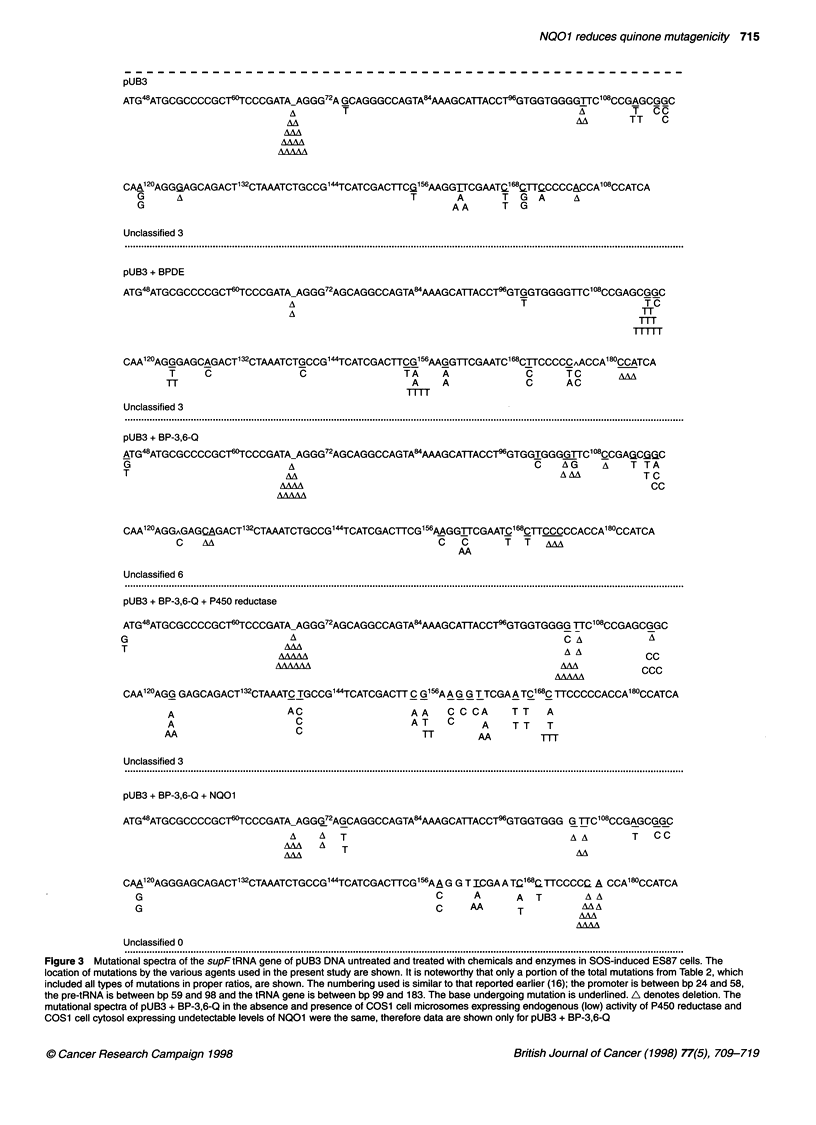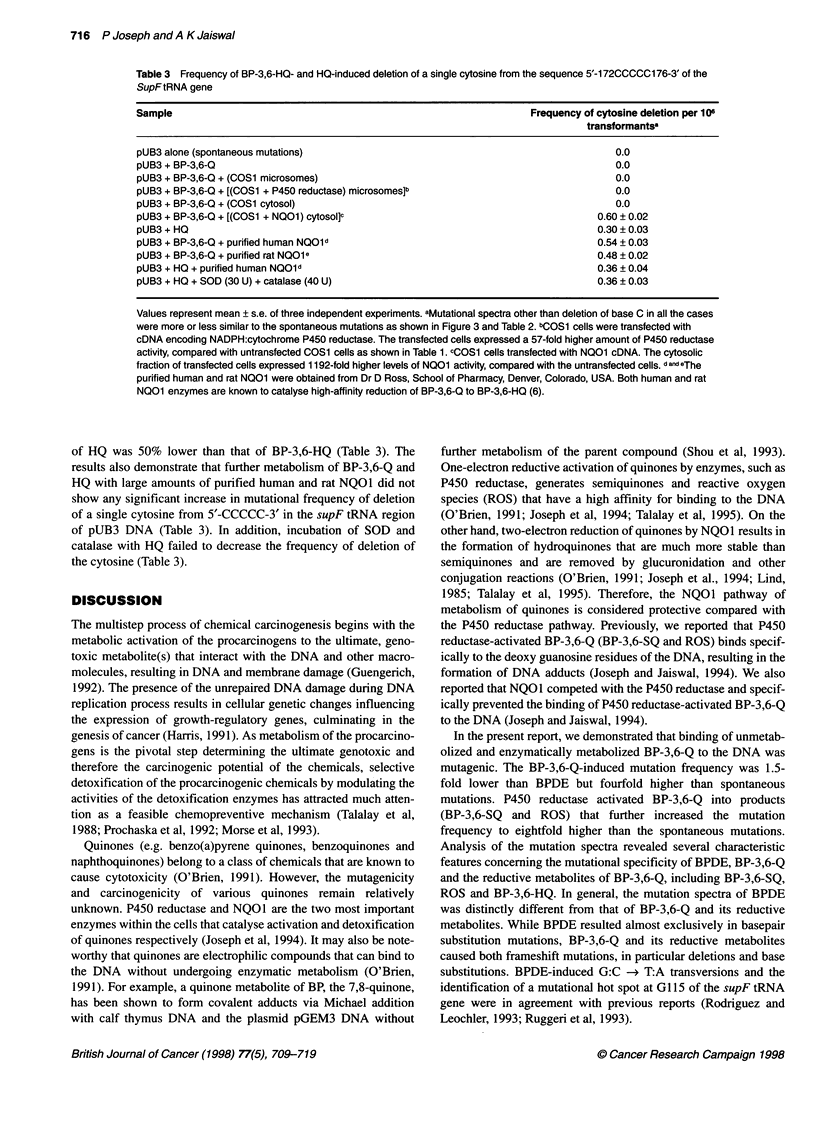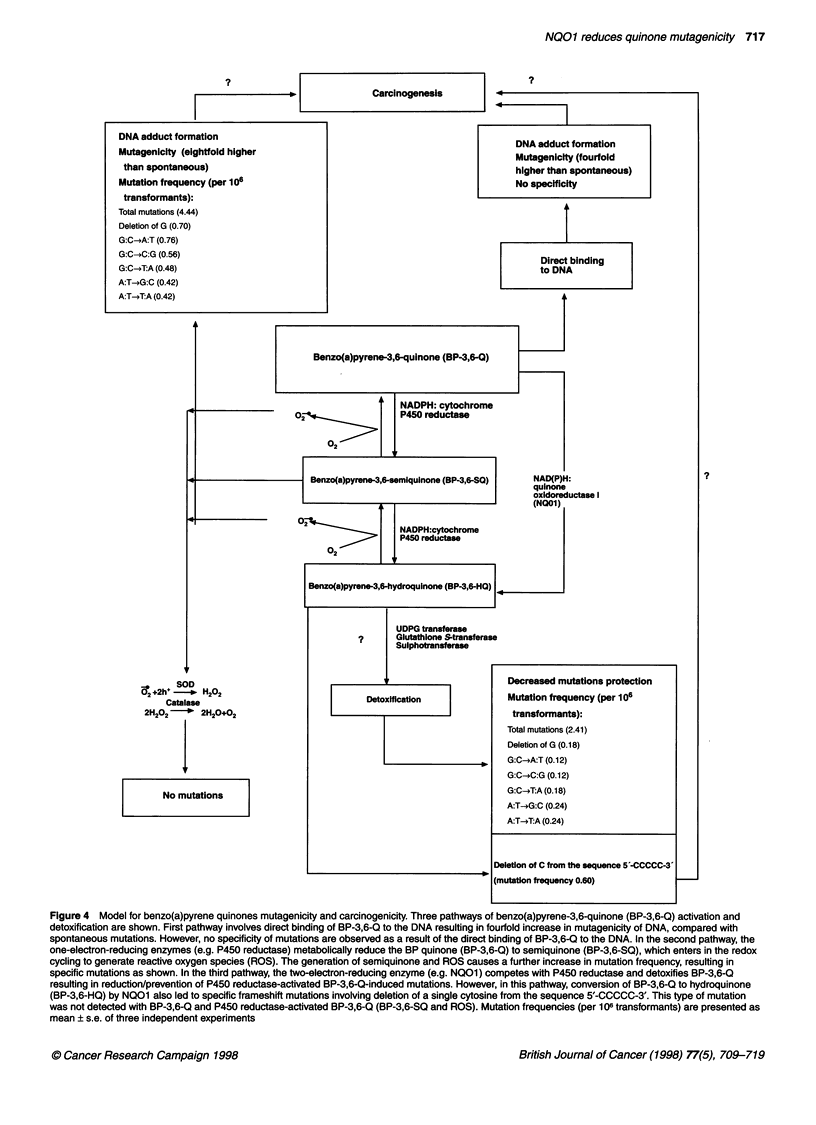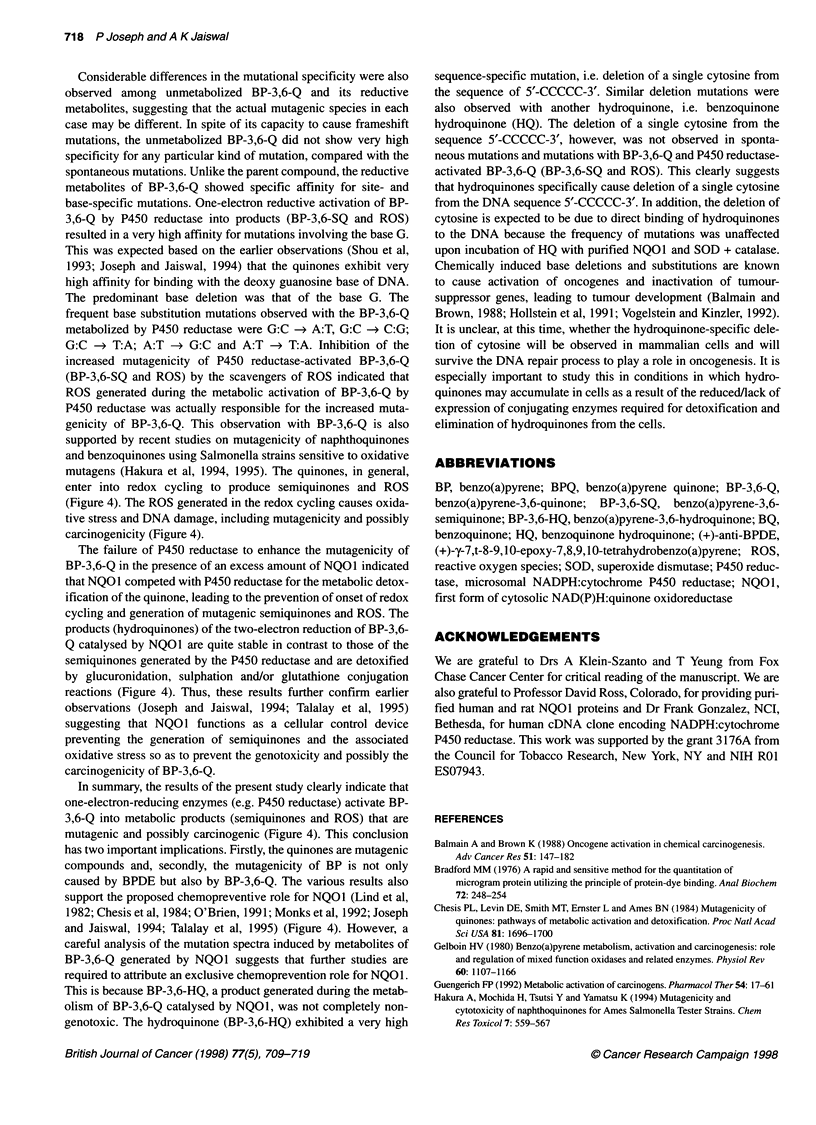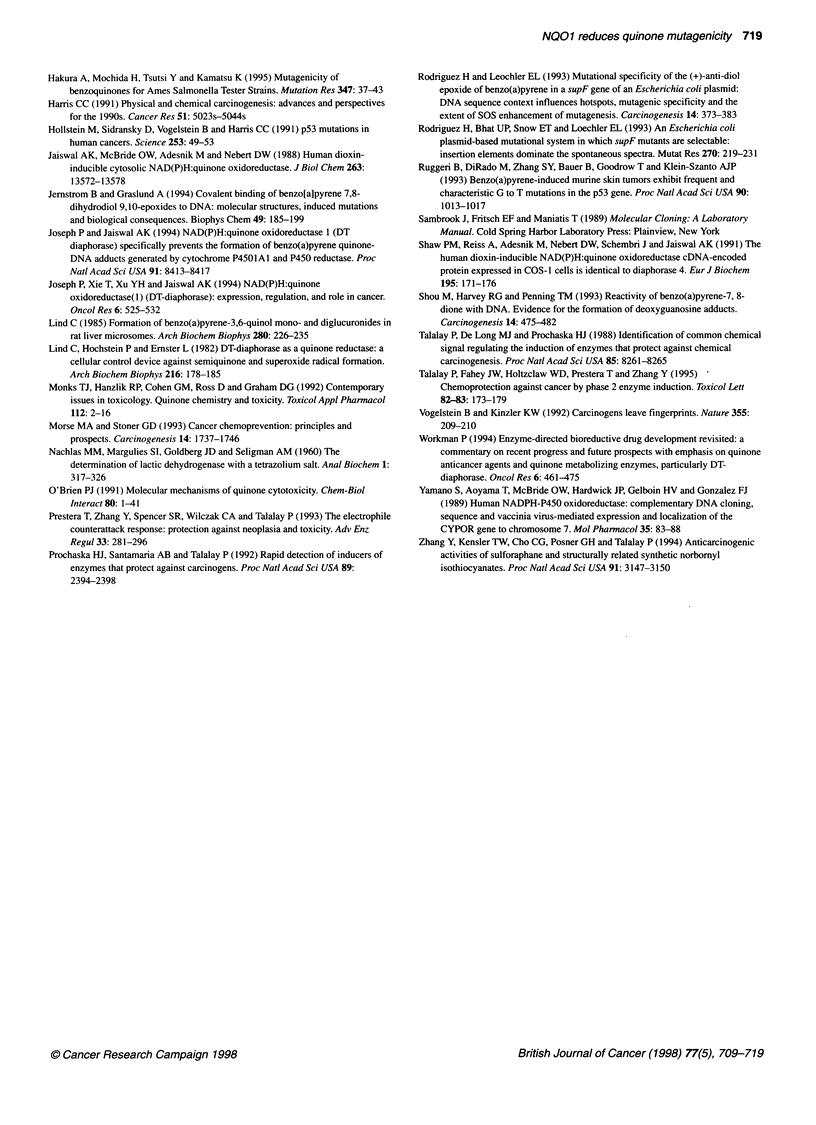# NAD(P)H:quinone oxidoreductase 1 reduces the mutagenicity of DNA caused by NADPH:P450 reductase-activated metabolites of benzo(a)pyrene quinones.

**DOI:** 10.1038/bjc.1998.117

**Published:** 1998-03

**Authors:** P. Joseph, A. K. Jaiswal

**Affiliations:** Department of Pharmacology, Fox Chase Cancer Center, Philadelphia, PA 19111, USA.

## Abstract

The role of microsomal NADPH:cytochrome P450 reductase (P450 reductase) and cytosolic NAD(P)H:quinone oxidoreductase 1 (NQO1 or DT-diaphorase) in the mutagenicity of benzo(a)pyrene-3,6-quinone (BP-3,6-Q) was studied using supF tRNA gene as the mutational target. pUB3 carrying the supF tRNA gene upon transformation into the Escherichia coli ES87 cells exhibited a spontaneous mutation frequency of 0.62 x 10(-6). Chemical modification of the pUB3 DNA with BP-3,6-Q caused a fourfold increase in the mutation frequency, compared with the spontaneous mutations. P450 reductase catalysed metabolic activation of BP-3,6-Q into reactive products (semiquinone and reactive oxygen species), which caused a further increase in the mutation frequency to eightfold over spontaneous mutations. Oxygen radical scavengers (SOD and catalase) blocked the P450 reductase-activated BP-3,6-Q-induced stimulation of mutations. This indicates that redox cycling of the semiquinone leading to the generation of reactive oxygen species (ROS) was directly responsible for the increased mutation frequency of P450 reductase-activated BP-3,6-Q. Analysis of the mutation spectra revealed that P450 reductase-activated BP-3,6-Q showed a significantly higher preference for frameshift mutations, particularly deletions, compared with the spontaneous mutations and the mutations generated by benzo(a)pyrene-7,8-dihydrodiol-9,10-epoxide (BPDE). The single most frequently observed mutation by P450 reductase-activated quinone (semiquinone + ROS) was deletion of a single guanosine. Among the base substitutions, G:C --> T:A, G:C --> A:T and G:C --> C:G were also noticed. Interestingly, NQO1 competed with P450 reductase and specifically prevented the P450 reductase-activated BP-3,6-Q-induced mutations. However, BP-hydroquinone (BP-3,6-HQ) generated during the metabolic reduction of BP-3,6-Q catalysed by NQO1 caused specific mutations involving the deletion of a single cytosine from the DNA sequence 5'-CCCCC-3' in supF tRNA gene at a significantly high frequency. A similar cytosine deletion was also observed with benzoquinone hydroquinone (HQ), indicating that the deletion of cytosine is associated with a hydroquinone class of compounds. These results suggest that: (1) quinones and P450 reductase-activated products of quinones (semiquinones and ROS) are mutagenic compounds; (2) the mutational spectra of quinones, semiquinones and hydroquinones differ from each other with respect to their mutational frequency and specificity; (3) NQO1 competes with P450 reductase and protects the cells from quinone mutagenicity; and (4) the NQO1 -metabolized quinones (hydroquinones), if not eliminated, cause specific mutations that are not observed with quinones and P450 reductase-activated quinones (semiquinones and ROS).


British Journal of Cancer (1998) 77(5), 709–719
© 1998 Cancer Research Campaign

### NAD(P)H:quinone oxidoreductase 1 reduces the mutagenicity of DNA caused by NADPH:P450 reductase-activated metabolites of benzo(a)pyrene quinones

**P Joseph and AK Jaiswal**

Department of Pharmacology, Fox Chase Cancer Center, 7701 Burholme Avenue, Philadelphia, PA 19111, USA

**Summary** The role of microsomal NADPH:cytochrome P450 reductase (P450 reductase) and cytosolic NAD(P)H:quinone oxidoreductase 1 (NQO1 or DT-diaphorase) in the mutagenicity of benzo(a)pyrene-3,6-quinone (BP-3,6-Q) was studied using *supF* tRNA gene as the mutational target. pUB3 carrying the *supF* tRNA gene upon transformation into the *Escherichia coli* ES87 cells exhibited a spontaneous mutation frequency of $0.62 \times 10^{-6}$. Chemical modification of the pUB3 DNA with BP-3,6-Q caused a fourfold increase in the mutation frequency, compared with the spontaneous mutations. P450 reductase catalysed metabolic activation of BP-3,6-Q into reactive products (semiquinone and reactive oxygen species), which caused a further increase in the mutation frequency to eightfold over spontaneous mutations. Oxygen radical scavengers (SOD and catalase) blocked the P450 reductase-activated BP-3,6-Q-induced stimulation of mutations. This indicates that redox cycling of the semiquinone leading to the generation of reactive oxygen species (ROS) was directly responsible for the increased mutation frequency of P450 reductase-activated BP-3,6-Q. Analysis of the mutation spectra revealed that P450 reductase-activated BP-3,6-Q showed a significantly higher preference for frameshift mutations, particularly deletions, compared with the spontaneous mutations and the mutations generated by benzo(a)pyrene-7,8-dihydrodiol-9,10-epoxide (BPDE). The single most frequently observed mutation by P450 reductase-activated quinone (semiquinone + ROS) was deletion of a single guanosine. Among the base substitutions, G:C → T:A, G:C → A:T and G:C → C:G were also noticed. Interestingly, NQO1 competed with P450 reductase and specifically prevented the P450 reductase-activated BP-3,6-Q-induced mutations. However, BP-hydroquinone (BP-3,6-HQ) generated during the metabolic reduction of BP-3,6-Q catalysed by NQO1 caused specific mutations involving the deletion of a single cytosine from the DNA sequence 5′-CCCCC-3′ in *supF* tRNA gene at a significantly high frequency. A similar cytosine deletion was also observed with benzoquinone hydroquinone (HQ), indicating that the deletion of cytosine is associated with a hydroquinone class of compounds. These results suggest that: (1) quinones and P450 reductase-activated products of quinones (semiquinones and ROS) are mutagenic compounds; (2) the mutational spectra of quinones, semiquinones and hydroquinones differ from each other with respect to their mutational frequency and specificity; (3) NQO1 competes with P450 reductase and protects the cells from quinone mutagenicity; and (4) the NQO1-metabolized quinones (hydroquinones), if not eliminated, cause specific mutations that are not observed with quinones and P450 reductase-activated quinones (semiquinones and ROS).

**Keywords:** NAD(P)H:quinone oxidoreductase 1; P450 reductase; benzo(a)pyrene-3,6-quinone; mutagenicity

The aetiology of human cancer is quite diverse and exposure to chemical carcinogens, particularly those present in the environment [e.g. benzo(a)pyrene (BP)] as contaminants, is responsible for the majority of incidences of human cancer. BP has been studied quite extensively as a prototype procarcinogen, whose involvement in carcinogenesis after DNA adduct formation and mutation has been fairly well documented (Gelboin, 1980; Jernstrom and Graslund, 1994). BP undergoes oxidative metabolism catalysed by cytochrome P450 1A1 (CYP 1A1) and NADPH:cytochrome P450 reductase (P450 reductase) to generate more than two dozen metabolites (Gelboin, 1980; O'Brien, 1991). The most studied metabolite of BP is benzo(a)pyrene-7,8-dihydrodiol-9,10-epoxide (BPDE). BPDE binds with the deoxyguanosine residues of DNA and causes mutations, preferably G:C → T:A transversions, that accumulate in growth-regulatory genes leading to carcinogenicity (Jernstrom and Graslund, 1994).

*Received 16 April 1997*
*Revised 14 April 1997*
*Accepted 22 July 1997*

*Correspondence to*: AK Jaiswal, Department of Pharmacology, Baylor College of Medicine, One Baylor Plaza, Houston, TX 77030, USA (present address)

Another important class of BP metabolites generated during its oxidative metabolism are the benzo(a)pyrene quinones (BP-3,6-Q, BP-1,6-Q and BP-6,12-Q) (Gelboin, 1980; O'Brien, 1991). Quinones, in general, are ubiquitously found in all aerobic plants and animals and are present in the environment as part of automobile exhaust, cigarette smoke and urban air particulates (O'Brien, 1991; Monks et al, 1992). Several compounds containing the quinone nucleus are extensively used as anti-tumour drugs (Workman, 1994). Because of their electrophilic nature and the very high redox potential, the quinones are highly toxic (O'Brien, 1991; Monks et al, 1992; Workman, 1994). Despite being present in significant amounts, thus posing a potential threat for extensive human exposure, and being highly reactive and toxic, the mechanisms underlying the toxicity of quinones, particularly with respect to their mutagenic and carcinogenic potential, are rather poorly understood. Earlier studies have shown that the fate of quinones inside the cells depends on the balance between phase I (cytochromes P450 and P450 reductase) enzymes that catalyse metabolic activation of quinones, leading to cellular damage, and phase II [NAD(P)H:quinone oxidoreductase 1, glutathione *S*-transferase and UDPG transferase) enzymes that catalyse metabolic detoxification of quinones, resulting in protection of the cells (Talalay et al, 1995). Accordingly, it has been proposed that induction of phase II enzymes by antioxidants, vitamins, isothiocyanates and related compounds leads to protection of cells against adverse effects of exposure to quinones and their precursors (Prestera et al, 1993; Zhang et al, 1994; Talalay et al, 1995). Recent studies in our laboratory on metabolic activation and detoxification of BPQs (BP-3,6-Q, BP-1,6-Q and BP-6,12-Q) provided evidence at the molecular level in support of the above hypothesis (Joseph and Jaiswal, 1994). Accordingly, the one-electron reduction of BPQ by P450 reductase resulted in the generation of benzo(a)pyrene semiquinones (BPSQ) and reactive oxygen species (ROS). The metabolites of BPQ (BPSQ and ROS) bind with the deoxyguanosine residues of the DNA, as evident from the generation of BPQ (metabolites)–DNA adducts. On the other hand, NQO1 competed with P450 reductase and catalysed two-electron reduction and detoxification of BPQ, thus protecting the cells from the generation of BPSQ, ROS and the corresponding DNA adducts.

In the present report, we have extended our studies on the mutagenicity of the unmetabolized quinones and P450 reductase-activated quinones (semiquinones and ROS) using BP-3,6-Q as the model quinone and using the *supF* suppressor tRNA gene of the *E. coli* plasmid pUB3 as the mutational target to understand the genotoxic basis for the mutagenic and carcinogenic potential of quinones. We also extended our studies to determine whether NQO1 exclusively reduces/prevents the mutagenicity of semiquinone and ROS or whether it also stimulates mutagenicity of quinones. The various results obtained by us demonstrate that P450 reductase-activated BPQ (BPSQ and ROS) binding to the DNA results in mutations (predominantly base deletions and substitutions). We also demonstrate that these mutations are caused as a result of the binding of ROS to the DNA. We further demonstrate that NQO1 reduces/prevents the mutations caused by P450 reductase-activated BPQ. In addition, we demonstrate that NQO1 catalyses the conversion of BPQ to BP hydroquinone (BPHQ), which causes specific mutations involving deletion of a single cytosine from the sequence 5′-CCCCC-3′ at a very high frequency, which was not observed with BPQ and P450 reductase-activated BPQ (BPSQ and ROS). We further demonstrate that deletion of a single cytosine from the sequence 5′-CCCCC-3′ is specifically associated with the hydroquinone class of compounds. This is because, in addition to BP-3,6-HQ, benzoquinone hydroquinone (HQ) also causes similar mutations at high frequency. The quantitative and qualitative aspects of mutations due to BPQ as well as its one- and two-electron reductive metabolites are compared with those due to BPDE and the possible implications are discussed.

### MATERIALS AND METHODS

### Materials

The *E. coli* strain ES87 and the plasmid pUB3 carrying the mutational target *supF* suppressor tRNA were obtained as a generous gift from Dr E Loechler (Boston University, Boston, MA, USA). DH5-α cells were purchased from Gibco-BRL (Gaithersburg, MD, USA). Ingredients of the media used to grow the bacterial cells and to select the mutants were purchased from Difco Laboratories (Detroit, MI, USA). All other reagents used in the experiments were of the highest purity available commercially. The DNA sequencing kit version 2.0 was purchased from USB Corporation, Cleveland, OH, USA. +(–)*anti*-BPDE and BP-3,6-quinone were purchased from the Chemical Carcinogen Repository of the National Cancer Institute (Kansas City, MO, USA). Benzoquinone hydroquinone (HQ) was purchased from Sigma Chemical Company, St Louis, MO, USA.

### Construction of pMT2-cDNA recombinant plasmids, transient transfection and enzyme assays

Human cDNAs encoding microsomal P450 reductase and cytosolic NQO1 have been cloned and sequenced (Jaiswal et al, 1988; Yamano et al, 1989). cDNAs for P450 reductase and NQO1 were separately subcloned into the unique *Eco*R1 site of the expression vector pMT2 (Shaw et al, 1991; Joseph and Jaiswal, 1994). The pMT2, pMT2-P450 reductase and pMT2-NQO1 plasmids were transiently transfected into monkey kidney COS1 cells in separate experiments by the DEAE-dextran and chloroquine method to over-express the respective proteins/activity (Shaw et al, 1991; Joseph and Jaiswal, 1994). The transfected COS1 cells were harvested, homogenized and subcellular-fractionated by procedures as described (Joseph and Jaiswal, 1994). The whole homogenate and microsomal and cytosolic fractions were analysed for P450 reductase and NQO1 activities using methods as described (Joseph and Jaiswal, 1994). Protein contents in the various fractions were estimated using Bradford's method (Bradford, 1976). The cytosolic and microsomal fractions of transfected COS1 cells were used as the source of P450 reductase and NQO1 enzymes, respectively, in incubation mixtures to study the role of these enzymes in metabolic reductive activation or detoxification of BP-3,6-Q, as determined by analysis of mutations in the *supF* tRNA region of plasmid pUB3.

### ES87 cells and pUB3 system to study mutations caused by BPDE and BP-3,6-Q. Adduction of pUB3 DNA with BPDE, BP-3,6-Q and hydroquinone pUB3 plasmid DNA, free from RNA and protein, was prepared using the Qiagen plasmid preparation kit (Qiagen, CA, USA) and further purified using phenol–chloroform extraction and ethanol precipitation (Sambrook et al, 1989). For the DNA adduct formation, 10 μg of pUB3 DNA was incubated with 100 ng of BPDE in a total volume of 200 μl of Tris buffer, pH 7.4, in the dark for 2 h. In the case of BP-3,6-Q-pUB3 DNA adducts, 10 μM BP-3,6-Q and 10 μg of the plasmid DNA were incubated either with the cytosolic NQO1 (15 μg of total protein) or with the microsomal P450 reductase (30 μg of total protein) and the co-factors required for the enzymes to metabolize BP-3,6-Q (Joseph and Jaiswal, 1994). In the experiments involving the use of oxygen-free radical scavengers, superoxide dismutase (SOD) and catalase were added into the reaction mixture. The reaction mixture (200 μl total volume) was incubated at 37°C for 2 h in the dark. The optimum conditions required for the reductive metabolism of BP-3,6-Q catalysed by the enzymes were obtained from preliminary experiments. At the end of the incubation period, 25 μl of the reaction mixture was withdrawn and used to estimate the amount of the cofactors (NADH in the case of NQO1 and NADPH in the case of P450 reductase) that remained unoxidized using the INT reagent (Nachlas et al, 1960). pUB3 DNA was isolated and purified from the remaining reaction mixture using phenol–chloroform extraction and ethanol precipitation (Sambrook et al, 1989). The DNA was dried briefly under

© Cancer Research Campaign 1998

vacuum, resuspended in Tris-EDTA buffer and used to transform competent ES87 cells. In similar experiments, BP-3,6-Q was replaced with 15 μM hydroquinone solubilized in ethanol. In related experiments, purified rat and human NQO1 enzymes were included with hydroquinone for adduction with pUB3; this was to study the role of NQO1 in further metabolism of hydroquinone and its effect on hydroquinone-induced mutagenicity.

### Transformation of competent ES87 cells and selection of the mutants

Competent ES87 cells were prepared and transformed either with the control pUB3 DNA or that adducted with the BP metabolites, according to the published procedures (Rodriguez and Leochler, 1993; Rodriguez et al, 1993). SOS induced (140 J $m^{-2}$) and uninduced ES87 cells were transformed with 1 μg of the pUB3 DNA by electroporation using the Gene Pulser (Bio-Rad, CA, USA) at 2.5 kV, 250 uF and 200 ohms according to the manufacturer's instructions. The transformants were allowed to recover in SOC medium for 1 h and plated on lactose minimal plates (LM plates) after diluting appropriately to select the mutants growing as large, blue colonies. The mutants were further screened by a second round of selection on LM plates followed by melibiose minimum (MM) plates, as described earlier (Rodriguez et al, 1993). A small portion of the transformants was diluted and plated on LB plates containing ampicillin to determine the total number of transformants. Mutation frequency (MF) was calculated on the basis of the number of mutants growing on the MM plates and the total number of the ampicillin-resistant transformants growing on the LB plates. DNA was isolated from the mutant colonies growing on the MM plates and used to transform competent DH5-α cells. Double-stranded DNA free of contaminating RNA and protein was isolated from the DH5-α cells and was analysed for mutations of the target *supF* tRNA gene by DNA sequencing. DNA sequencing was performed with T7 DNA polymerase using the sequenase kit version 2.0, according to the manufacturer's instructions. It may be noteworthy that every mutation was picked up from a single transformation to avoid the generation of siblings of individual mutations.

### Competition between P450 reductase and NQO1 for BP-3,6-Q metabolism and mutation

In order to assess the capacity of P450 reductase and NQO1 to compete for the reductive metabolism of BP-3,6-Q and to result in mutation, the microsomes possessing P450 reductase and the cytosol possessing NQO1 activity from the COS1 cells transfected separately with the corresponding cDNAs were used. The enzyme samples were mixed in different proportions and used in the incubation mixture containing BP-3,6-Q, pUB3 DNA and the cofactors required for the reductive metabolism of BP-3,6-Q. In different experiments, either the amount of P450 reductase or that of NQO1 was changed by keeping that of the other one constant. At the end of the incubation period, DNA was isolated and used to transform competent, SOS-induced ES87 cells, and the mutation frequency was determined as described above.

### RESULTS

### Activities of microsomal P450 reductase and NQO1 in untransfected and transfected monkey kidney COS1 cells

The microsomal P450 reductase and cytosolic NQO1 activities in the untransfected and transfected monkey kidney COS1 cells are shown in Table 1. The untransfected COS1 cells possessed an undetectable amount of endogenous NQO1 and a very low amount of P450 reductase activity (Table 1; Joseph and Jaiswal, 1994). The pMT2-P450 reductase-transfected COS1 cells produced 57-fold higher P450 reductase activity than the untransfected control cells. Similarly, the pMT2-NQO1-transfected COS1 cells expressed 1192-fold higher NQO1 activity than the untransfected COS1 cells. These higher activities of P450 reductase and NQO1 allowed us to use the corresponding subcellular fractions (microsomes for P450 reductase and cytosol for NQO1) for metabolic reduction of BP-3,6-Q to study mutagenicity of quinones and the role of these enzymes in quinone mutagenicity.

### Mutation frequency

In general, mutation frequency, either spontaneous or that due to the chemical modification of the pUB3 DNA, was higher (approximately two- to threefold) in the SOS-induced cells than in those that were not subjected to SOS induction (data not shown). With the exception of the quantitative difference in mutation frequency, no other change in response to the SOS induction of the ES87 cells was noticed. Therefore, only those results obtained with the SOS-induced ES87 cells are presented hereafter. In addition, the incubation of pUB3 DNA with BP-3,6-Q showed similar frequency and type of mutations as those observed with pUB3 + BP-3,6-Q mixed and incubated with untransfected COS1 cell microsomes or cytosol

**Table 1** Enzyme activities in untransfected and cDNA-transfected monkey kidney COS1 cells

| Cells | Microsomal cytochrome P450 reductase (nmol of cytochrome c reduced $min^{-1}$ $mg^{-1}$ protein) | Cytosolic NQO1 (μmol of 2,6-dichlorophenolindophenol reduced $min^{-1}$ $mg^{-1}$ protein) |
|---|---|---|
| COS1 (untransfected) | 16.34 ± 1.12 | 0.031 ± 0.002 |
| COS1 + pMT2-human P450 reductase | 925.00 ± 23.91 | 0.034 ± 0.001 |
| COS1 + pMT2-human NQO1 | 17.22 ± 1.25 | 36.940 ± 1.020 |

Cytosolic NQO1 activity was absent in microsomal fractions. Similarly, microsomal P450 reductase was absent in cytosolic fractions.

© Cancer Research Campaign 1998

**Figure 1** **A** Mutation frequency of *supF* tRNA gene after transformation into SOS-induced ES87 cells. Control pUB3 DNA or DNA adducted with the benzo(a)pyrene metabolites as described in the text were used to transform SOS-induced ES87 cells. Mutation frequency was determined as described in the text. Data represent the mean ± s.e. of three independent experiments. (**B**) Role of microsomal P450 reductase in BP-3,6-Q mutagenicity. pUB3 DNA was incubated with DMSO (pUB3) or BP-3,6-Q or BP-3,6-Q + COS1 cell microsomes (expressing endogenous levels of P450 reductase) or BP-3,6-Q + COS1 cell microsomal fractions expressing 57-fold higher-levels of cDNA-derived P450 reductase, compared with untransfected COS1 cells expressing endogenous levels of P450 reductase. In many cases purified SOD and/or catalase were combined with the COS1 cell microsomal fraction containing high levels of P450 reductase before incubation with pUB3 and BP-3,6-Q. After the incubations, the pUB3 DNA was isolated from the reaction mixture and used to transform SOS-induced competent ES87 cells, and the mutation frequency was determined as described in the text. It may be noteworthy that the mutation frequency observed with pUB3 + BP-3,6-Q was similar to the mutation frequency observed with pUB3 + BP-3,6-Q + COS1 microsomes from untransfected cells containing low levels of P450 reductase. Therefore, data are shown only for pUB3 + BP-3,6-Q. Data represent the mean ± s.e. of three independent experiments expressing endogenous (low to undetectable) levels of P450 reductase and NQO1. Therefore, in all the experiments, we have shown only results that were obtained with pUB3 + BP-3,6-Q.

The chemical modification of the pUB3 DNA with either BPDE or BP-3,6-Q resulted in significant increases in the mutation frequency compared with that of the spontaneous mutations with the control (untreated) pUB3 DNA (Fig 1A). The treatment of pUB3 DNA with BPDE and BP-3,6-Q resulted in a sevenfold and a fourfold increase, respectively, in the mutation frequency, compared with the background mutation frequency. The metabolic reductive activation of BP-3,6-Q by P450 reductase (one-electron-reducing enzyme) further increased the mutation frequency of BP-3,6-Q by approximately twofold, compared with the unmetabolized BP-3,6-Q, and by 8.2-fold, compared with the frequency of spontaneous mutations (Figure 1B). Interestingly, the pUB3 mutations induced by P450 reductase-activated BP-3,6-Q were eliminated/prevented by superoxide dismutase (SOD) and catalase (Figure 1B). Thirty units of SOD alone was less effective than 40 units of catalase and a combination of 7.5 units of SOD with 10 units of catalase (Figure 1B). The P450 reductase-activated BP-3,6-Q-induced mutations in pUB3 were also significantly reduced as a result of the inclusion of NQO1 with P450 reductase in the metabolism of BP-3,6-Q (Figure 2A). Increasing the concentration of NQO1 with a constant concentration of P450 reductase showed a NQO1 concentration-dependent decrease in pUB3 mutations caused by BP-3,6-Q (Figure 2A). In a similar experiment, increasing the concentration of P450 reductase with a constant concentration of NQO1 increased the pUB3 mutations caused by BP-3,6-Q (Figure 2B). The slow increase in mutation frequency with increasing concentration of P450 reductase may be due to the presence of large quantities of NQO1, which is known to compete with P450 reductase and to detoxify quinones (Figure 2B). In another related experiment, the increase in the concentration of NQO1 caused steady but less significant decreases in BP-3,6-Q (unmetabolized)-induced pUB3 mutations (Figure 2C). It may be noteworthy that incubation of BPDE with P450 reductase and of NQO1 individually and in combination had no effect on the mutation frequency, compared with BPDE alone (data not shown).

 © Cancer Research Campaign 1998

**Figure 2** Competition between cytochrome P450 reductase and NQO1 to metabolize benzo(a)pyrene-3,6-quinone and its role in quinone mutagenicity. **(A)** pUB3 DNA was incubated with DMSO or BP-3,6-Q, a constant amount (30 µg of protein) of COS1 cell microsomes expressing high levels of P450 reductase and varying amounts (µg of protein) of COS1 cytosolic extract expressing very high levels of NQO1. **(B)** pUB3 DNA was incubated with DMSO or BP-3,6-Q, a constant amount (µg of protein) of COS1 cytosolic extract expressing very high levels of NQO1 and varying amounts (µg of protein) of COS1 cell microsomes expressing high levels of P450 reductase. **(C)** pUB3 DNA was incubated with DMSO or BP-3,6-Q and varying amounts (µg of protein) of COS1 cytosolic extract expressing very high levels of NQO1. In each case, after the incubation, the pUB3 DNA was isolated from the reaction mixture and was used to transform SOS-induced ES87 cells. The mutation frequency was determined as described in the text. The data represent the mean ± s.e. of three independent experiments

### Mutation spectra

The mutation frequencies of the various treatments of pUB3 DNA are shown in Table 2. The location of mutations by the various agents used in the present study are shown in Figure 3. It is noteworthy that only a portion of the total mutations from Table 2, which includes all types of mutations in proper ratios, are shown in Figure 3. The analysis of mutation frequencies and the mutation spectra of the different treatments showed that they were distinctly different, suggesting the chemical specificity of mutations (Table 2 and Figure 3).

Among spontaneous mutations (pUB3 alone), the single most abundant mutation was the deletion of 51 bp in the region extending from bp 68 to bp 119 of the *supF* tRNA gene. BPDE exhibited a very high specificity for the G:C → T:A transversion, which accounted for 51% of all the mutations. It was found that the mutational frequency of BPDE-induced G:C → T:A transversions was 46-fold higher than spontaneous mutations. Fifty per cent of BPDE-induced G:C → T:A transversions was associated with the base G at nucleotide position 115 of the *supF* tRNA gene of the pUB3 (Figure 3). Frameshift mutations were very rare in the case of BPDE.

Unlike BPDE, the BP-3,6-Q and its metabolites frequently caused frameshift mutations involving base deletions (Table 2). In addition, they also caused base substitutions. The unmetabolized BP-3,6-Q showed a several-fold higher frequency of similar mutations, as observed in spontaneous mutations (Table 2). They did not exhibit any strict mutational specificity, and therefore no mutational hot spots were identifiable in this case. However, P450 reductase-activated BP-3,6-Q showed characteristic mutational specificity (Table 2 and Figure 3). BP-3,6-Q reductively activated by the P450 reductase showed a high preference for mutations involving the base G (52% of all the mutations, mutational frequency of 1.54). The most frequently observed mutations were the deletion of base G from the DNA sequence 5′-GGGG-3′ between nucleotide positions 102–105 of the *supF* tRNA (mutation frequency 0.70) and G:C → A:T substitutions (mutation frequency 0.76) (Table 2 and Figure 3; lane pUB3 + BP-3,6-Q+P450 reductase). In addition, the P450 reductase-activated metabolites of BP-3,6-Q also caused other base substitution mutations, including G:C → C:G (frequency 0.56), G:C → T:A (mutation frequency 0.48), A:T → G:C (mutation frequency 0.42) and A:T → T:A (mutation frequency 0.42) (Table 2 and Figure 3). Interestingly, in the case of BP-3,6-Q metabolized by NQO1, the mutation frequency of deletion of base G and all kinds of base substitutions were significantly reduced compared with the P450 reductase-activated BP-3,6-Q (Table 2 and Figure 3). Accordingly, the frequency of the deletion of base G was reduced from 0.70 in the case of P450 reductase-activated BP-3,6-Q to 0.18 in the case of NQO1-metabolized BP-3,6-Q (Table 2). The frequency of base substitutions was also significantly reduced in the presence of NQO1; these included G:C → T:A from 0.48 to 0.18, G:C → A:T from 0.76 to 0.12, G:C → C:G from 0.56 to 0.12, A:T → G:C from 0.42 to 0.24 and A:T → T:A from 0.42 to 0.24 (Table 2). The only mutational specificity observed in the case of BP-3,6-Q metabolized by NQO1 was a frameshift mutation involving deletion of a single cytosine from the DNA sequence 5′-CCCCC-3′ between the

© Cancer Research Campaign 1998

**Table 2** Mutations detected in the *supF* tRNA gene of pUB3, both unadducted (spontaneous) and adducted with BPQ and BPQ metabolites, after transformation into SOS-induced ES87 cells

| | pUB3 | pUB3 +BPDE | pUB3 +BP-3,6-Q | pUB3 +BP-3,6-Q + reductase | pUB3 +BP-3,6-Q + NQO1 |
|---|---|---|---|---|---|
| Total number of mutant colonies selected from 65 × $10^6$ transformed ES87 cells | 40 (0.62) | 176 (4.4) | 110 (2.76) | 178 (4.44) | 96 (2.41) |
| Base substitutions | | | | | |
| G:C → T:A | 3 (0.05) | **92 (2.3)** | 8 (0.20) | **19 (0.48)** | 7 (0.18) |
| G:C → A:T | 2 (0.03) | **28 (0.7)** | 8 (0.20) | **31 (0.76)** | 5 (0.12) |
| G:C → C:G | 5 (0.08) | 9 (0.23) | 8 (0.20) | **22 (0.56)** | 5 (0.12) |
| A:T → G:C | 2 (0.03) | 9 (0.23) | 8 (0.20) | 17 (0.42) | 10 (0.24) |
| A:T → C:G | 0 (0.00) | 5 (0.12) | 6 (0.14) | 6 (0.14) | 5 (0.12) |
| A:T → T:A | 6 (0.09) | 0 (0.00) | 8 (0.20) | 17 (0.42) | 10 (0.24) |
| Deletions | | | | | |
| G | 1 (0.02) | 0 (0.00) | 3 (0.07) | **28 (0.70)** | 7 (0.18) |
| A single C from DNA sequence 5′-CCCCC-3′ | 0 (0.00) | 0 (0.00) | 0 (0.00) | 0 (0.00) | **24 (0.60)** |
| C | 0 (0.00) | 0 (0.00) | 3 (0.07) | 0 (0.00) | 0 (0.00) |
| T | 3 (0.05) | 0 (0.00) | 6 (0.14) | 6 (0.14) | 7 (0.18) |
| A | 1 (0.02) | 0 (0.00) | 0 (0.00) | 0 (0.00) | 5 (0.12) |
| AA | 0 (0.00) | 0 (0.00) | 0 (0.00) | 3 (0.07) | 0 (0.00) |
| CCA | 0 (0.00) | 5 (0.12) | 0 (0.00) | 0 (0.00) | 0 (0.00) |
| CCC | 0 (0.00) | 0 (0.00) | 3 (0.07) | 0 (0.00) | 0 (0.00) |
| GGGTTCCCG | 0 (0.00) | 0 (0.00) | 3 (0.07) | 0 (0.00) | 0 (0.00) |
| bp 68–119 | 15 (0.23) | 9 (0.23) | 33 (0.84) | 39 (0.97) | 17 (0.042) |
| bp 103–111 | 0 (0.00) | 0 (0.00) | 3 (0.07) | 0 (0.00) | 0 (0.00) |
| Insertions | | | | | |
| C | 0 (0.00) | 9 (0.23) | 3 (0.07) | 0 (0.00) | 0 (0.00) |
| Unclassified | 3 (0.045) | 14 (0.36) | 17 (0.42) | 5 (0.14) | 0 (0.00) |
| Total number of mutations detected | 41 | 180 | 120 | 193 | 102 |

pUB3 DNA was treated with dimethyl sulphoxide (DMSO) or BPDE or BP-3,6-Q in the absence and presence of the enzymes. The adducted DNA was isolated, cleaned, precipitated and used to transform SOS-induced *E. coli* ES87 cells. Mutants were isolated by procedures as described (16–17). None of the mutations represent siblings because every mutation was picked from a single transformation. Note that the total number of mutations detected is different to the total number of mutant colonies isolated. This is because, in a few cases, we detected more than one mutation. Numbers in parentheses represent the mutation frequency (× $10^{-6}$ or $10^6$ transformants). The mutants were isolated in five independent experiments. The mutation frequency (× $10^{-6}$) is the average of the three most reliable experiments. The specific and significant mutations are shown in bold numbers. Unclassified mutations included insertion of large fragments of DNA that were not sequenced.

nucleotides 172 and 176 at a mutation frequency of 0.60 (Table 2). This deletion of a single cytosine from the DNA sequence of 5′-CCCCC-3′ was not observed in spontaneous mutations or mutations caused by BPDE, unmetabolized BP-3,6-Q or P450 reductase-activated metabolic products of BP-3,6-Q (semiquinone and ROS). The results from previous studies and those from the present report were used to generate a model to understand the role of various enzymes in the metabolic activation, detoxification, mutagenicity and possibly carcinogenicity of BP-3,6-Q (Figure 4).

### Hydroquinone mutagenicity

The mutational spectra of BP-3,6-HQ (generated as a result of the two-electron reduction of BP-3,6-Q catalysed by NQO1) was very interesting, as described above. It caused specific deletion of a single cytosine from the sequence 5′-CCCCC-3′ in the *supF* tRNA gene at a high frequency. This type of deletion mutation was not observed in spontaneous mutations and the mutations caused by BP-3,6-Q and P450 reductase-catalysed metabolites of BP-3,6-Q (BP-3,6-SQ and ROS) (Table 3). These observations raise three very important questions. First, do other hydroquinones also cause similar mutations involving deletion of a single cytosine from the DNA sequence 5′-CCCCC-3′ as observed with BP-3,6-HQ? Second, does BP-3,6-HQ undergo further metabolism catalysed by NQO1 to generate unidentified metabolites that cause deletion mutation of a single cytosine? Third, does BP-3,6-HQ undergo auto-oxidation by atmospheric oxygen to generate ROS that causes specific deletion of a single cytosine from the sequence 5′-CCCCC-3′? Several mutagenesis experiments were performed with BP-3,6-HQ and a second hydroquinone, benzoquinone hydroquinone (HQ), to address these questions (Table 3). HQ, such as BP-3,6-HQ, caused specific deletion of a single cytosine from the sequence 5′-CCCCC-3′ of the *supF* tRNA gene. However, the mutation frequency of cytosine deletion as a result

 © Cancer Research Campaign 1998

**Figure 3** Mutational spectra of the *supF* tRNA gene of pUB3 DNA untreated and treated with chemicals and enzymes in SOS-induced ES87 cells. The location of mutations by the various agents used in the present study are shown. It is noteworthy that only a portion of the total mutations from Table 2, which included all types of mutations in proper ratios, are shown. The numbering used is similar to that reported earlier (16); the promoter is between bp 24 and 58, the pre-tRNA is between bp 59 and 98 and the tRNA gene is between bp 99 and 183. The base undergoing mutation is underlined. △ denotes deletion. The mutational spectra of pUB3 + BP-3,6-Q in the absence and presence of COS1 cell microsomes expressing endogenous (low) activity of P450 reductase and COS1 cell cytosol expressing undetectable levels of NQO1 were the same, therefore data are shown only for pUB3 + BP-3,6-Q

Table 3 Frequency of BP-3,6-HQ- and HQ-induced deletion of a single cytosine from the sequence 5′-172CCCCC176-3′ of the *SupF* tRNA gene

| Sample | Frequency of cytosine deletion per $10^6$ transformants[a] |
|---|---|
| pUB3 alone (spontaneous mutations) | 0.0 |
| pUB3 + BP-3,6-Q | 0.0 |
| pUB3 + BP-3,6-Q + (COS1 microsomes) | 0.0 |
| pUB3 + BP-3,6-Q + [(COS1 + P450 reductase) microsomes][b] | 0.0 |
| pUB3 + BP-3,6-Q + (COS1 cytosol) | 0.0 |
| pUB3 + BP-3,6-Q + [(COS1 + NQO1) cytosol][c] | 0.60 ± 0.02 |
| pUB3 + HQ | 0.30 ± 0.03 |
| pUB3 + BP-3,6-Q + purified human NQO1[d] | 0.54 ± 0.03 |
| pUB3 + BP-3,6-Q + purified rat NQO1[e] | 0.48 ± 0.02 |
| pUB3 + HQ + purified human NQO1[d] | 0.36 ± 0.04 |
| pUB3 + HQ + SOD (30 U) + catalase (40 U) | 0.36 ± 0.03 |

Values represent mean ± s.e. of three independent experiments. [a]Mutational spectra other than deletion of base C in all the cases were more or less similar to the spontaneous mutations as shown in Figure 3 and Table 2. [b]COS1 cells were transfected with cDNA encoding NADPH:cytochrome P450 reductase. The transfected cells expressed a 57-fold higher amount of P450 reductase activity, compared with untransfected COS1 cells as shown in Table 1. [c]COS1 cells transfected with NQO1 cDNA. The cytosolic fraction of transfected cells expressed 1192-fold higher levels of NQO1 activity, compared with the untransfected cells. [d and e]The purified human and rat NQO1 were obtained from Dr D Ross, School of Pharmacy, Denver, Colorado, USA. Both human and rat NQO1 enzymes are known to catalyse high-affinity reduction of BP-3,6-Q to BP-3,6-HQ (6).

of HQ was 50% lower than that of BP-3,6-HQ (Table 3). The results also demonstrate that further metabolism of BP-3,6-Q and HQ with large amounts of purified human and rat NQO1 did not show any significant increase in mutational frequency of deletion of a single cytosine from 5′-CCCCC-3′ in the *supF* tRNA region of pUB3 DNA (Table 3). In addition, incubation of SOD and catalase with HQ failed to decrease the frequency of deletion of the cytosine (Table 3).

### DISCUSSION

The multistep process of chemical carcinogenesis begins with the metabolic activation of the procarcinogens to the ultimate, genotoxic metabolite(s) that interact with the DNA and other macromolecules, resulting in DNA and membrane damage (Guengerich, 1992). The presence of the unrepaired DNA damage during DNA replication process results in cellular genetic changes influencing the expression of growth-regulatory genes, culminating in the genesis of cancer (Harris, 1991). As metabolism of the procarcinogens is the pivotal step determining the ultimate genotoxic and therefore the carcinogenic potential of the chemicals, selective detoxification of the procarcinogenic chemicals by modulating the activities of the detoxification enzymes has attracted much attention as a feasible chemopreventive mechanism (Talalay et al, 1988; Prochaska et al, 1992; Morse et al, 1993).

Quinones (e.g. benzo(a)pyrene quinones, benzoquinones and naphthoquinones) belong to a class of chemicals that are known to cause cytotoxicity (O'Brien, 1991). However, the mutagenicity and carcinogenicity of various quinones remain relatively unknown. P450 reductase and NQO1 are the two most important enzymes within the cells that catalyse activation and detoxification of quinones respectively (Joseph et al, 1994). It may also be noteworthy that quinones are electrophilic compounds that can bind to the DNA without undergoing enzymatic metabolism (O'Brien, 1991). For example, a quinone metabolite of BP, the 7,8-quinone, has been shown to form covalent adducts via Michael addition with calf thymus DNA and the plasmid pGEM3 DNA without further metabolism of the parent compound (Shou et al, 1993). One-electron reductive activation of quinones by enzymes, such as P450 reductase, generates semiquinones and reactive oxygen species (ROS) that have a high affinity for binding to the DNA (O'Brien, 1991; Joseph et al, 1994; Talalay et al, 1995). On the other hand, two-electron reduction of quinones by NQO1 results in the formation of hydroquinones that are much more stable than semiquinones and are removed by glucuronidation and other conjugation reactions (O'Brien, 1991; Joseph et al., 1994; Lind, 1985; Talalay et al, 1995). Therefore, the NQO1 pathway of metabolism of quinones is considered protective compared with the P450 reductase pathway. Previously, we reported that P450 reductase-activated BP-3,6-Q (BP-3,6-SQ and ROS) binds specifically to the deoxy guanosine residues of the DNA, resulting in the formation of DNA adducts (Joseph and Jaiswal, 1994). We also reported that NQO1 competed with the P450 reductase and specifically prevented the binding of P450 reductase-activated BP-3,6-Q to the DNA (Joseph and Jaiswal, 1994).

In the present report, we demonstrated that binding of unmetabolized and enzymatically metabolized BP-3,6-Q to the DNA was mutagenic. The BP-3,6-Q-induced mutation frequency was 1.5-fold lower than BPDE but fourfold higher than spontaneous mutations. P450 reductase activated BP-3,6-Q into products (BP-3,6-SQ and ROS) that further increased the mutation frequency to eightfold higher than the spontaneous mutations. Analysis of the mutation spectra revealed several characteristic features concerning the mutational specificity of BPDE, BP-3,6-Q and the reductive metabolites of BP-3,6-Q, including BP-3,6-SQ, ROS and BP-3,6-HQ. In general, the mutation spectra of BPDE was distinctly different from that of BP-3,6-Q and its reductive metabolites. While BPDE resulted almost exclusively in basepair substitution mutations, BP-3,6-Q and its reductive metabolites caused both frameshift mutations, in particular deletions and base substitutions. BPDE-induced G:C → T:A transversions and the identification of a mutational hot spot at G115 of the *supF* tRNA gene were in agreement with previous reports (Rodriguez and Leochler, 1993; Ruggeri et al, 1993).

 © Cancer Research Campaign 1998

**Figure 4** Model for benzo(a)pyrene quinones mutagenicity and carcinogenicity. Three pathways of benzo(a)pyrene-3,6-quinone (BP-3,6-Q) activation and detoxification are shown. First pathway involves direct binding of BP-3,6-Q to the DNA resulting in fourfold increase in mutagenicity of DNA, compared with spontaneous mutations. However, no specificity of mutations are observed as a result of the direct binding of BP-3,6-Q to the DNA. In the second pathway, the one-electron-reducing enzymes (e.g. P450 reductase) metabolically reduce the BP quinone (BP-3,6-Q) to semiquinone (BP-3,6-SQ), which enters in the redox cycling to generate reactive oxygen species (ROS). The generation of semiquinone and ROS causes a further increase in mutation frequency, resulting in specific mutations as shown. In the third pathway, the two-electron-reducing enzyme (e.g. NQO1) competes with P450 reductase and detoxifies BP-3,6-Q resulting in reduction/prevention of P450 reductase-activated BP-3,6-Q-induced mutations. However, in this pathway, conversion of BP-3,6-Q to hydroquinone (BP-3,6-HQ) by NQO1 also led to specific frameshift mutations involving deletion of a single cytosine from the sequence 5′-CCCCC-3′. This type of mutation was not detected with BP-3,6-Q and P450 reductase-activated BP-3,6-Q (BP-3,6-SQ and ROS). Mutation frequencies (per $10^6$ transformants) are presented as mean ± s.e. of three independent experiments

© Cancer Research Campaign 1998 

Considerable differences in the mutational specificity were also observed among unmetabolized BP-3,6-Q and its reductive metabolites, suggesting that the actual mutagenic species in each case may be different. In spite of its capacity to cause frameshift mutations, the unmetabolized BP-3,6-Q did not show very high specificity for any particular kind of mutation, compared with the spontaneous mutations. Unlike the parent compound, the reductive metabolites of BP-3,6-Q showed specific affinity for site- and base-specific mutations. One-electron reductive activation of BP-3,6-Q by P450 reductase into products (BP-3,6-SQ and ROS) resulted in a very high affinity for mutations involving the base G. This was expected based on the earlier observations (Shou et al, 1993; Joseph and Jaiswal, 1994) that the quinones exhibit very high affinity for binding with the deoxy guanosine base of DNA. The predominant base deletion was that of the base G. The frequent base substitution mutations observed with the BP-3,6-Q metabolized by P450 reductase were G:C → A:T, G:C → C:G; G:C → T:A; A:T → G:C and A:T → T:A. Inhibition of the increased mutagenicity of P450 reductase-activated BP-3,6-Q (BP-3,6-SQ and ROS) by the scavengers of ROS indicated that ROS generated during the metabolic activation of BP-3,6-Q by P450 reductase was actually responsible for the increased mutagenicity of BP-3,6-Q. This observation with BP-3,6-Q is also supported by recent studies on mutagenicity of naphthoquinones and benzoquinones using Salmonella strains sensitive to oxidative mutagens (Hakura et al, 1994, 1995). The quinones, in general, enter into redox cycling to produce semiquinones and ROS (Figure 4). The ROS generated in the redox cycling causes oxidative stress and DNA damage, including mutagenicity and possibly carcinogenicity (Figure 4).

The failure of P450 reductase to enhance the mutagenicity of BP-3,6-Q in the presence of an excess amount of NQO1 indicated that NQO1 competed with P450 reductase for the metabolic detoxification of the quinone, leading to the prevention of onset of redox cycling and generation of mutagenic semiquinones and ROS. The products (hydroquinones) of the two-electron reduction of BP-3,6-Q catalysed by NQO1 are quite stable in contrast to those of the semiquinones generated by the P450 reductase and are detoxified by glucuronidation, sulphation and/or glutathione conjugation reactions (Figure 4). Thus, these results further confirm earlier observations (Joseph and Jaiswal, 1994; Talalay et al, 1995) suggesting that NQO1 functions as a cellular control device preventing the generation of semiquinones and the associated oxidative stress so as to prevent the genotoxicity and possibly the carcinogenicity of BP-3,6-Q.

In summary, the results of the present study clearly indicate that one-electron-reducing enzymes (e.g. P450 reductase) activate BP-3,6-Q into metabolic products (semiquinones and ROS) that are mutagenic and possibly carcinogenic (Figure 4). This conclusion has two important implications. Firstly, the quinones are mutagenic compounds and, secondly, the mutagenicity of BP is not only caused by BPDE but also by BP-3,6-Q. The various results also support the proposed chemopreventive role for NQO1 (Lind et al, 1982; Chesis et al, 1984; O'Brien, 1991; Monks et al, 1992; Joseph and Jaiswal, 1994; Talalay et al, 1995) (Figure 4). However, a careful analysis of the mutation spectra induced by metabolites of BP-3,6-Q generated by NQO1 suggests that further studies are required to attribute an exclusive chemoprevention role for NQO1. This is because BP-3,6-HQ, a product generated during the metabolism of BP-3,6-Q catalysed by NQO1, was not completely non-genotoxic. The hydroquinone (BP-3,6-HQ) exhibited a very high sequence-specific mutation, i.e. deletion of a single cytosine from the sequence of 5′-CCCCC-3′. Similar deletion mutations were also observed with another hydroquinone, i.e. benzoquinone hydroquinone (HQ). The deletion of a single cytosine from the sequence 5′-CCCCC-3′, however, was not observed in spontaneous mutations and mutations with BP-3,6-Q and P450 reductase-activated BP-3,6-Q (BP-3,6-SQ and ROS). This clearly suggests that hydroquinones specifically cause deletion of a single cytosine from the DNA sequence 5′-CCCCC-3′. In addition, the deletion of cytosine is expected to be due to direct binding of hydroquinones to the DNA because the frequency of mutations was unaffected upon incubation of HQ with purified NQO1 and SOD + catalase. Chemically induced base deletions and substitutions are known to cause activation of oncogenes and inactivation of tumour-suppressor genes, leading to tumour development (Balmain and Brown, 1988; Hollstein et al, 1991; Vogelstein and Kinzler, 1992). It is unclear, at this time, whether the hydroquinone-specific deletion of cytosine will be observed in mammalian cells and will survive the DNA repair process to play a role in oncogenesis. It is especially important to study this in conditions in which hydroquinones may accumulate in cells as a result of the reduced/lack of expression of conjugating enzymes required for detoxification and elimination of hydroquinones from the cells.

### ABBREVIATIONS

BP, benzo(a)pyrene; BPQ, benzo(a)pyrene quinone; BP-3,6-Q, benzo(a)pyrene-3,6-quinone; BP-3,6-SQ, benzo(a)pyrene-3,6-semiquinone; BP-3,6-HQ, benzo(a)pyrene-3,6-hydroquinone; BQ, benzoquinone; HQ, benzoquinone hydroquinone; (+)-anti-BPDE, (+)-γ-7,t-8-9,10-epoxy-7,8,9,10-tetrahydrobenzo(a)pyrene; ROS, reactive oxygen species; SOD, superoxide dismutase; P450 reductase, microsomal NADPH:cytochrome P450 reductase; NQO1, first form of cytosolic NAD(P)H:quinone oxidoreductase

### ACKNOWLEDGEMENTS

We are grateful to Drs A Klein-Szanto and T Yeung from Fox Chase Cancer Center for critical reading of the manuscript. We are also grateful to Professor David Ross, Colorado, for providing purified human and rat NQO1 proteins and Dr Frank Gonzalez, NCI, Bethesda, for human cDNA clone encoding NADPH:cytochrome P450 reductase. This work was supported by the grant 3176A from the Council for Tobacco Research, New York, NY and NIH R01 ES07943.

### REFERENCES

Balmain A and Brown K (1988) Oncogene activation in chemical carcinogenesis. *Adv Cancer Res* **51**: 147–182

Bradford MM (1976) A rapid and sensitive method for the quantitation of microgram protein utilizing the principle of protein-dye binding. *Anal Biochem* **72**: 248–254

Chesis PL, Levin DE, Smith MT, Ernster L and Ames BN (1984) Mutagenicity of quinones: pathways of metabolic activation and detoxification. *Proc Natl Acad Sci USA* **81**: 1696–1700

Gelboin HV (1980) Benzo(a)pyrene metabolism, activation and carcinogenesis: role and regulation of mixed function oxidases and related enzymes. *Physiol Rev* **60**: 1107–1166

Guengerich FP (1992) Metabolic activation of carcinogens. *Pharmacol Ther* **54**: 17–61

Hakura A, Mochida H, Tsutsi Y and Yamatsu K (1994) Mutagenicity and cytotoxicity of naphthoquinones for Ames Salmonella Tester Strains. *Chem Res Toxicol* **7**: 559–567

© Cancer Research Campaign 1998

Hakura A, Mochida H, Tsutsi Y and Kamatsu K (1995) Mutagenicity of benzoquinones for Ames Salmonella Tester Strains. *Mutation Res* **347**: 37–43

Harris CC (1991) Physical and chemical carcinogenesis: advances and perspectives for the 1990s. *Cancer Res* **51**: 5023s–5044s

Hollstein M, Sidransky D, Vogelstein B and Harris CC (1991) p53 mutations in human cancers. *Science* **253**: 49–53

Jaiswal AK, McBride OW, Adesnik M and Nebert DW (1988) Human dioxin-inducible cytosolic NAD(P)H:quinone oxidoreductase. *J Biol Chem* **263**: 13572–13578

Jernstrom B and Graslund A (1994) Covalent binding of benzo[a]pyrene 7,8-dihydrodiol 9,10-epoxides to DNA: molecular structures, induced mutations and biological consequences. Biophys Chem **49**: 185–199

Joseph P and Jaiswal AK (1994) NAD(P)H:quinone oxidoreductase 1 (DT diaphorase) specifically prevents the formation of benzo(a)pyrene quinone-DNA adducts generated by cytochrome P4501A1 and P450 reductase. *Proc Natl Acad Sci USA* **91**: 8413–8417

Joseph P, Xie T, Xu YH and Jaiswal AK (1994) NAD(P)H:quinone oxidoreductase(1) (DT-diaphorase): expression, regulation, and role in cancer. *Oncol Res* **6**: 525–532

Lind C (1985) Formation of benzo(a)pyrene-3,6-quinol mono- and diglucuronides in rat liver microsomes. *Arch Biochem Biophys* **280**: 226–235

Lind C, Hochstein P and Ernster L (1982) DT-diaphorase as a quinone reductase: a cellular control device against semiquinone and superoxide radical formation. *Arch Biochem Biophys* **216**: 178–185

Monks TJ, Hanzlik RP, Cohen GM, Ross D and Graham DG (1992) Contemporary issues in toxicology. Quinone chemistry and toxicity. *Toxicol Appl Pharmacol* **112**: 2–16

Morse MA and Stoner GD (1993) Cancer chemoprevention: principles and prospects. *Carcinogenesis* **14**: 1737–1746

Nachlas MM, Margulies SI, Goldberg JD and Seligman AM (1960) The determination of lactic dehydrogenase with a tetrazolium salt. *Anal Biochem* **1**: 317–326

O'Brien PJ (1991) Molecular mechanisms of quinone cytotoxicity. *Chem-Biol Interact* **80**: 1–41

Prestera T, Zhang Y, Spencer SR, Wilczak CA and Talalay P (1993) The electrophile counterattack response: protection against neoplasia and toxicity. *Adv Enz Regul* **33**: 281–296

Prochaska HJ, Santamaria AB and Talalay P (1992) Rapid detection of inducers of enzymes that protect against carcinogens. *Proc Natl Acad Sci USA* **89**: 2394–2398

Rodriguez H and Leochler EL (1993) Mutational specificity of the (+)-anti-diol epoxide of benzo(a)pyrene in a *supF* gene of an *Escherichia coli* plasmid: DNA sequence context influences hotspots, mutagenic specificity and the extent of SOS enhancement of mutagenesis. *Carcinogenesis* **14**: 373–383

Rodriguez H, Bhat UP, Snow ET and Loechler EL (1993) An *Escherichia coli* plasmid-based mutational system in which *supF* mutants are selectable: insertion elements dominate the spontaneous spectra. Mutat Res **270**: 219–231

Ruggeri B, DiRado M, Zhang SY, Bauer B, Goodrow T and Klein-Szanto AJP (1993) Benzo(a)pyrene-induced murine skin tumors exhibit frequent and characteristic G to T mutations in the p53 gene. *Proc Natl Acad Sci USA* **90**: 1013–1017

Sambrook J, Fritsch EF and Maniatis T (1989) *Molecular Cloning: A Laboratory Manual*. Cold Spring Harbor Laboratory Press: Plainview, New York

Shaw PM, Reiss A, Adesnik M, Nebert DW, Schembri J and Jaiswal AK (1991) The human dioxin-inducible NAD(P)H:quinone oxidoreductase cDNA-encoded protein expressed in COS-1 cells is identical to diaphorase 4. *Eur J Biochem* **195**: 171–176

Shou M, Harvey RG and Penning TM (1993) Reactivity of benzo(a)pyrene-7, 8-dione with DNA. Evidence for the formation of deoxyguanosine adducts. *Carcinogenesis* **14**: 475–482

Talalay P, De Long MJ and Prochaska HJ (1988) Identification of common chemical signal regulating the induction of enzymes that protect against chemical carcinogenesis. *Proc Natl Acad Sci USA* **85**: 8261–8265

Talalay P, Fahey JW, Holtzclaw WD, Prestera T and Zhang Y (1995) Chemoprotection against cancer by phase 2 enzyme induction. *Toxicol Lett* **82–83**: 173–179

Vogelstein B and Kinzler KW (1992) Carcinogens leave fingerprints. *Nature* **355**: 209–210

Workman P (1994) Enzyme-directed bioreductive drug development revisited: a commentary on recent progress and future prospects with emphasis on quinone anticancer agents and quinone metabolizing enzymes, particularly DT-diaphorase. *Oncol Res* **6**: 461–475

Yamano S, Aoyama T, McBride OW, Hardwick JP, Gelboin HV and Gonzalez FJ (1989) Human NADPH-P450 oxidoreductase: complementary DNA cloning, sequence and vaccinia virus-mediated expression and localization of the CYPOR gene to chromosome 7. *Mol Pharmacol* **35**: 83–88

Zhang Y, Kensler TW, Cho CG, Posner GH and Talalay P (1994) Anticarcinogenic activities of sulforaphane and structurally related synthetic norbornyl isothiocyanates. *Proc Natl Acad Sci USA* **91**: 3147–3150

© Cancer Research Campaign 1998